# Supplementary material for: The genome sequence of the Blood-vein moth, Timandra comae Schmidt, 1931
Source: Wellcome Open Res. 2024 Mar 1;9:100. [Version 1] doi: 10.12688/wellcomeopenres.21088.1 (PMC11391194; doi:10.12688/wellcomeopenres.21088.1)
Supplement: Peer review history

# Reviewer report: Reviewer response for version 1

**DOI:** 10.21956/wellcomeopenres.23328.r98736

**Published:** 2024-10-01

**Authors:** Marko Mutanen

**Affiliations:** 1 University of Oulu, Oulu, Finland

This article publishes a genome sequence for a looper (Geometridae) moth *Timandra comae*. *Timandra comae* belong to the subfamily Sterrhinae and within it a group that appears somewhat distinct from other Sterrhinae in Europe, except for a very close *T. griseata* which is so close to *T. comae* that their status as distinct species have sometimes been questioned. Publishing the genome therefore helps in studying this question is the future. It should be noted that the name of *T. griseata* is misspelled in the article's Background section (as griselata).

 The analyzed specimen is a male.

 The article make statements of 1) the two above-mentioned species being separable in their DNA barcodes and 2) that *T. griseata* is Scandinavian. Both cannot be true, because the cluster of griseata is found in UK by many specimens in BOLD.

 The article undoubtedly lists reported food plants of T. comae as mentioned in literature, but there tend to be lots of errors, and in this case I would strongly question Atriplex spp. being among the food plants.

 As shown in the snailplot graph of Figure 2 and other graphs, the published genome is clearly of high standard. This is indicated e.g. by high BUSCO coverage (only 1.2% missing) and scaffold statistics that indicate high quality.

Are sufficient details of methods and materials provided to allow replication by others?

Yes

Is the rationale for creating the dataset(s) clearly described?

Yes

Are the datasets clearly presented in a useable and accessible format?

Yes

Are the protocols appropriate and is the work technically sound?

Yes

Reviewer Expertise:

Lepidoptera diversity, taxonomy, phylogenetics, DNA barcoding, phylogenomics

I confirm that I have read this submission and believe that I have an appropriate level of expertise to confirm that it is of an acceptable scientific standard.

# Reviewer report: Reviewer response for version 1

**DOI:** 10.21956/wellcomeopenres.23328.r98735

**Published:** 2024-09-11

**Authors:** Kay Lucek

**Affiliations:** 1 University of Neuchâtel, Neuchâtel, Switzerland

The authors present the chromosome level genome assembly of a male specimen of the blood vein moth *Timandra comae*. The assembly consists of 31 chromosomes including the Z chromosome. The assembly is highly complete as revealed by the high BUSCO score but not fully phased. Sequencing and genome assembly follow the current state of the art and use established methods.

 Overall, the presented assembly will be of great value to study genome architecture as well as genome size evolution in Lepidoptera. I particularly appreciate the biological background given for this species, where the reference genome provides the opportunity to study the range expansion of this species in the UK and elsewhere.

Are sufficient details of methods and materials provided to allow replication by others?

Yes

Is the rationale for creating the dataset(s) clearly described?

Yes

Are the datasets clearly presented in a useable and accessible format?

Yes

Are the protocols appropriate and is the work technically sound?

Yes

Reviewer Expertise:

Speciation genomics

I confirm that I have read this submission and believe that I have an appropriate level of expertise to confirm that it is of an acceptable scientific standard.

# Reviewer report: Reviewer response for version 1

**DOI:** 10.21956/wellcomeopenres.23328.r96299

**Published:** 2024-09-09

**Authors:** Niclas Backström

**Affiliations:** 1 Uppsala University, Uppsala, Sweden

Minor comments:

 Background, 2nd paragraph. "its having" sounds a bit odd - consider editing

 Background, 4th paragraph. Unclear what "chromosomally complete" really means. Consider editing.

 GSReport, 2nd paragraph. Here it is stated that the assembly contains 39 scaffolds. The number is the same as in table 1, but in Figure 2, 3 and 4 it is stated that the assembly consists of 40 scaffolds. Maybe I misunderstood something, but check if it is correct.

Are sufficient details of methods and materials provided to allow replication by others?

Yes

Is the rationale for creating the dataset(s) clearly described?

Yes

Are the datasets clearly presented in a useable and accessible format?

Yes

Are the protocols appropriate and is the work technically sound?

Yes

Reviewer Expertise:

Evolutionary genomics

I confirm that I have read this submission and believe that I have an appropriate level of expertise to confirm that it is of an acceptable scientific standard.
